# Metabolomics Contributions to the Discovery of Prostate Cancer Biomarkers

**DOI:** 10.3390/metabo9030048

**Published:** 2019-03-08

**Authors:** Nuria Gómez-Cebrián, Ayelén Rojas-Benedicto, Arturo Albors-Vaquer, José Antonio López-Guerrero, Antonio Pineda-Lucena, Leonor Puchades-Carrasco

**Affiliations:** 1 Drug Discovery Unit, Instituto de Investigación Sanitaria La Fe, Valencia 46026, Spain; ngomez@cipf.es (N.G.-C.); ayelen_rojas@iislafe.es (A.R.-B.); arturo_albors@iislafe.es (A.A.-V.); pineda_ant@gva.es (A.P.-L.); 2 Joint Research Unit in Clinical Metabolomics, Centro de Investigación Príncipe Felipe/Instituto de Investigación Sanitaria La Fe, Valencia 46012, Spain; 3 Laboratory of Molecular Biology, Fundación Instituto Valenciano de Oncología, Valencia 46009, Spain; jalopez@fivo.org

**Keywords:** metabolomics, metabolism, prostate cancer, biomarker, early diagnosis, prognosis

## Abstract

Prostate cancer (PCa) is one of the most frequently diagnosed cancers and a leading cause of death among men worldwide. Despite extensive efforts in biomarker discovery during the last years, currently used clinical biomarkers are still lacking enough specificity and sensitivity for PCa early detection, patient prognosis, and monitoring. Therefore, more precise biomarkers are required to improve the clinical management of PCa patients. In this context, metabolomics has shown to be a promising and powerful tool to identify novel PCa biomarkers in biofluids. Thus, changes in polyamines, tricarboxylic acid (TCA) cycle, amino acids, and fatty acids metabolism have been reported in different studies analyzing PCa patients’ biofluids. The review provides an up-to-date summary of the main metabolic alterations that have been described in biofluid-based studies of PCa patients, as well as a discussion regarding their potential to improve clinical PCa diagnosis and prognosis. Furthermore, a summary of the most significant findings reported in these studies and the connections and interactions between the different metabolic changes described has also been included, aiming to better describe the specific metabolic signature associated to PCa.

## 1. Introduction

Prostate cancer (PCa) is the second most frequently diagnosed cancer and represents the fifth leading cause of death in men [1]. In 2018, new cases of PCa were estimated to account for over 1.3 million, and 359.000 PCa-associated deaths were expected worldwide [1]. PCa is a hormone-dependent tumor characterized by an extremely variable clinical course, ranging from an indolent condition to a rapid progression into an aggressive phenotype that disseminates and metastasizes to the lymph nodes and bones. Moreover, there is a current lack of reliable and reproducible assays to identify tumors destined to remain indolent. Thus, stratifying PCa patients into different risk phenotypes at time of diagnosis is still a major clinical challenge.

Nowadays, PCa screening tests rely on the determination of prostate-specific antigen (PSA) serum levels and digital rectal examination (DRE). Based on the results of these screening tests, trans-rectal ultrasound (TRUS)-guided prostate biopsy is performed to confirm diagnosis when necessary. However, these tests suffer from a number of limitations and do not provide enough information to enable a precise discrimination between indolent and aggressive tumors. While PSA provides high sensitivity and low specificity for PCa diagnosis, (TRUS)-guided prostate biopsy has been associated with high false negative rates due to the high degree of PCa inter- and intra-heterogeneity [2]. Moreover, even the recently updated histopathology-based estimation of the Gleason Score (GS), the current clinical gold standard for assessing the risk of PCa metastasis and prognosis, exhibits limitations [3]. During the last years, many research studies have focused on the identification of molecular biomarkers that could help to improve early diagnosis and risk stratification of PCa patients [4,5,6,7]. Among them, a potential biomarker, that has been evaluated in combination with PSA levels, is the non-coding transcript PCA3 (overexpressed in >95% of PCa). The quantification of PCA3 levels in urine has shown improvement, when combined with PSA, in PCa detection [8], although no optimal cut-off for urinary PCA3 levels has been established for maximizing clinical benefit while avoiding overdiagnosis [9]. Another potential biomarker is the TMPRSS2:ERG fusion transcript [10], that is being evaluated as a potential diagnostic and therapeutic target associated with PCa invasion [11]. Despite being 100% indicative of PCa [12], it is only detected in 50% of PCa cases [13]. In summary, although intense efforts have been devoted to the discovery and development of new PCa biomarkers, there still exists an unmet clinical need to identify accurate PCa biomarkers for early diagnosis, prognosis and monitoring of PCa patients, both in terms of sensitivity and specificity [14,15].

Moreover, additional clinically robust biomarkers able to differentiate between indolent and aggressive PCa are urgently needed. In this context, several metabolomics studies have been carried out to attempt the characterization of a specific PCa metabolic profile, with the ultimate goal of identifying potential metabolic biomarkers that could improve the clinical management of PCa patients [16,17,18,19].

## 2. Cancer and Metabolic Reprogramming: Metabolomics Opportunities

The metabolic profile is closely associated with the pathophysiological condition of an individual. In particular, the metabolic composition can be strongly influenced, both from a qualitative and quantitative point of view, as a result of pathological processes or in the presence of specific drug treatments [20]. These changes can provide useful clues for the characterization of biomarkers associated with the onset and progression of diseases, as well as with the prediction of the response to therapeutic interventions.

Different studies, linking significant metabolic alterations and cancer onset and progression, have been extensively described since Warburg’s pioneering studies [21]. The metabolic rewiring associated with the neoplastic processes is the result of mutations in specific oncogenes and tumor suppressors, leading to the activation of different signaling pathways and transcriptional networks [22]. Furthermore, it is well known that neoplastic processes have a strong influence on gene expression, cellular differentiation and tumor microenvironment [23,24]. Metabolites represent the end products of biochemical pathways, and the concentrations of these compounds are extremely sensitive to different alterations. At the molecular level, the progression of cancer involves multiple alterations in metabolic pathways that are specifically required for cancer cells to survive [23]. Interestingly, cancer cells exhibit different metabolic phenotypes [25,26]. Thus, some tumors preferentially use aerobic glycolysis to proliferate [27], while others rely on glutaminolysis [28], or one-carbon metabolism [29]. There are also tumors that benefit from the utilization of several of these metabolic routes at the same time [25,26,28].

In this context, metabolomics, that relies on the systematic analysis of low-molecular-weight metabolites present in biological samples, provides an accurate and complementary approach for getting a better understanding of the biochemical alterations responsible for the onset and progression of neoplastic processes, thus offering new opportunities for biomarker discovery in complex diseases [30]. Metabolomics studies offer a holistic view of the biochemical processes that could contribute to getting a deeper insight into the molecular alterations underlying pathological processes. This information could significantly improve the opportunities to identify clinically relevant biomarkers for the diagnosis and prognosis of different pathological processes, including PCa.

## 3. Metabolomics and PCa

The ultimate goal of metabolomics is to measure and identify as many metabolites as possible, ideally obtaining a complete overview of the metabolome. Metabolomics can provide an accurate description of the phenotype of an individual because it represents the final step of the omics cascade. The analysis of metabolic changes associated with specific biochemical pathways offers unprecedented opportunities for identifying the molecular mechanisms of complex diseases. Taken into consideration the limitations of current diagnostic procedures, this information could result in the characterization of specific and novel disease biomarkers [31].

At the analytical level, these studies are extremely challenging [32,33]. The complexity of the matrix to be examined (e.g., osmolarity, the presence of proteins, and inorganic salt concentration), the dynamic range of metabolites concentrations, and the vast chemical diversity of metabolite types (e.g., acidic, neutral, basic, lyophilic, and hydrophilic) greatly complicate the choice of analytical modality. However, a number of technical improvements have been introduced over the last few years. This has led to the development of a wide variety of analytical platforms that are currently used to characterize the metabolic content of biological samples [34,35,36]. The selection of the appropriate approach usually depends on the experimental objectives and the biological matrix. The detection of metabolites in cells, tissues or biofluids is usually carried out by either Nuclear Magnetic Resonance (NMR) spectroscopy or mass spectrometry (MS). In general, NMR spectroscopy, mostly ^1^H-NMR, and MS, particularly liquid chromatography (LC)-MS, are the two most important analytical platforms used in metabolomics studies.

PCa is a disease of great interest from a metabolomics perspective. A number of studies, focused on the characterization of the specific PCa metabolic phenotype using different experimental approaches, have been reported recently [37,38,39,40,41,42,43,44,45,46,47,48,49,50,51,52,53,54,55,56,57,58,59,60,61]. These studies have shown that healthy prostate cells are characterized by a decreased citrate oxidation and metabolism within the tricarboxylic acid (TCA) cycle, resulting in citrate accumulation [62] and the reliance on glucose oxidation for energy production [63]. Benign prostate cells accumulate zinc, resulting in the inhibition of the m-aconitase (ACO), the enzyme that catalyzes the isomerization of citrate in the TCA cycle [62]. However, when prostate cells undergo malignant transformation, their characteristic ability to accumulate zinc is lost, leading to the TCA activation. Furthermore, it has been shown that early PCa does not exhibit the Warburg effect [64], relying on lipids and other energetic molecules for energy production, but not on aerobic respiration [65,66]. In this context, it should be noted that several metabolic alterations have also been identified in PCa tissue compared with normal tissue, including an increase of choline [67] and sarcosine [68], and a decrease of polyamine and citrate levels [69,70]. Nevertheless, the clinical relevance of some of these changes remains controversial due to the contradictory results reported in different studies (e.g., alterations in sarcosine levels–further discussed in the following section).

Overall, the possibility to directly evaluate the metabolic phenotype of PCa patients offers a great potential from a clinical perspective. To this end, many metabolomics projects, based on the analysis of different biological samples, have been conducted over the last few years with a focus on the discovery of new biomarkers that could improve the clinical management of PCa patients (Table 1).

## 4. PCa Metabolic Biomarkers in Biofluids

Changes in the concentration of metabolites in biofluids are reflective of alterations in the physiological status of an individual. The metabolome, that is, the set of all metabolites present on a particular biological sample, represents the downstream end product of the omics cascade, and a closer approach to the phenotype. Therefore, metabolite signatures obtained from biofluids can be a useful approach for identifying non-invasive biomarkers and characterizing the molecular mechanisms associated with pathological conditions. The most widely used biofluids in PCa studies have been urine, serum and seminal fluid.

### 4.1. Urine Biomarkers

Urine samples offer some advantages for carrying out metabolomics studies since they can be collected non-invasively and have a less complex composition compared with other biofluids, thus facilitating the discovery of novel biomarkers [71]. However, the analysis of this biofluid has several limitations, including the presence of diluted urinary constituents and interferences between molecules [37,71], that can result in failing to detect underrepresented metabolites or to correctly identify the molecules. Despite these problems, different studies have discovered metabolic alterations in urine samples from PCa patients and evaluated their clinical utility as biomarkers for this neoplastic process.

Urine is anatomically close to the prostate, which explains why it has been extensively studied for metabolic biomarker discovery in PCa [37]. As shown in Table 1, most of these studies have aimed to identify metabolic dysregulations that could provide clinically relevant PCa biomarkers. Most of these studies focused on the characterization of the metabolic differences between urine samples from healthy individuals [38,39,40,41,42,43] or benign prostate hyperplasia (BPH) patients [37,44,45] and PCa patients. In general, they were performed using mass spectrometry (MS)-based metabolomics as an analytical platform (*n* = 8), and only one study was performed using NMR spectroscopy for the analysis of urine samples [44].

The study conducted by Liang et al., including the analysis of 233 healthy individuals and 236 PCa patients, highlighted the clinical utility of three metabolites: 5-hydroxy-l-tryptophan, hippurate, and glycocholic acid, as potential metabolic biomarkers for the early diagnosis of PCa (area under the curve, (AUC) > 0.95) [38]. A metabolite called 5-hydroxy-l-tryptophan is involved in tryptophan metabolism, a pathway that has been associated with the ability of several tumors to evade the antitumor immune response [72,73]. Another metabolite involved in this pathway, kynurenic acid, also exhibited a moderate diagnostic value (AUC = 0.62) in a study conducted by Gkotsos et al. for the detection of PCa using urine samples obtained after prostatic massage [39].

Another metabolite that has been extensively investigated as a potential biomarker of PCa is sarcosine. Sarcosine is an intermediate product in the synthesis and degradation of glycine. In 2009, Sreekumar et al. identified sarcosine as a promising PCa biomarker, being highly correlated with PCa progression and more detectable in the urine of PCa patients when compared with healthy individuals [68]. Similarly, Khan et al. reported in 2013 markedly elevated sarcosine levels in the urine sediments of PCa patients compared with controls [74]. In serum, Kumar et al. [46,47] also found increased sarcosine levels in PCa samples compared with healthy individuals. In these studies, it was shown that sarcosine, in combination with other metabolites, could accurately differentiate PCa patients from healthy individuals (accuracy = 90.2%) [47] and PCa from BPH patients (87.7% sensitivity and 85.5% specificity) [46]. Furthermore, the authors showed that metabolomics provided better predictions than serum PSA levels for the discrimination between PCa patients and healthy individuals as well as between PCa and BPH patients. However, the role of sarcosine as a metabolic biomarker for PCa diagnosis and prognosis remains controversial due to the contradictory results reported in further studies. In a case-control study conducted by Ankerst et al., the use of sarcosine as a biomarker for early PCa detection was investigated in serum samples of matched-age controls and PCa patients [75]. These authors reported no differences in sarcosine levels when comparing both groups. Furthermore, in another pilot study by Dereziński et al., where higher serum sarcosine levels were found in PCa patients when compared with the control group, no statistically significant differences were observed in urine samples [76]. Similarly, Pérez-Rambla et al. found elevated sarcosine levels in PCa patients when compared with BPH patients, although these alterations were not found to be statistically significant [44].

Beyond the alteration in sarcosine levels, Pérez-Rambla et al. also identified alterations in the urine levels of six metabolites that facilitated the discrimination of the metabolomic profile of PCa and BPH patients [44]. Among the characteristic changes, PCa patients showed decreased concentration of glycine, a metabolite involved in one-carbon metabolism and associated with cell transformation and tumorigenesis [77]. Interestingly, Struck-Lewicka et al. reported lower levels of this metabolite in urine samples from PCa patients when compared with a control group [40]. The overall results of this study showed alterations in the urine levels of metabolites associated with TCA cycle, purine, glucose, amino acid and urea metabolism in PCa patients. These findings are in agreement with those obtained by Fernández-Peralbo et al., where variations in the levels of 28 metabolites involved in amino acid, purine and pyrimidine, and tryptophan metabolism were also identified [41] when comparing PCa patients and healthy individuals. The results of this study led to a predictive model of high quality for the discrimination of these two groups (sensitivity = 88.4% sensitivity, specificity = 92.9%).

Metabolic changes have also been identified when comparing urine samples from low and high risk PCa patients. Heger et al. performed a study focused on the characterization of differences in protein expression levels between two different risk groups of PCa patients after radical prostatectomy (RP) [48]. The two experimental cohorts were divided based on the presence of positive (*n* = 15) or negative (*n* = 15) surgical margins. The analysis led to the identification of three proteins with different expression levels. Among them, the glycolytic enzyme lactate dehydrogenase C (LDHC), that plays a key role in metabolism, was detected at higher expression levels in PCa patients with positive surgical margins [48]. Beyond PCa, increased LDHC expression has also been observed in melanoma, lung and breast cancer [78]. Moreover, this enzyme has been shown to be involved in tumor invasion and migration in breast cancer [79].

A complementary approach, that has also been the focus of recent studies in the context of urinary alterations associated with PCa, is the analysis of extracellular vesicles (EV). The analysis of these particles still requires the optimization of methods for isolation and storage of urinary EV, as well as for the normalization of metabolite levels [80]. Nevertheless, in a preliminary study, Puhka et al. analyzed urine EV samples from three controls and three PCa patients, obtained before and after prostatectomy [42]. After normalization tests, decreased levels of glucuronate, D-ribose 5-phosphate and isobutyryl-L-carnitine were observed in pre-prostatectomy samples when compared with the healthy individuals and post-prostatectomy samples. In agreement with these results, Clos-García et al. also reported variations in carnitine-related metabolites when comparing urine EV samples from PCa (*n* = 31) and BPH (*n* = 14) patients [37]. In this study, changes in the expression levels of seven enzymes related to fatty acid, steroid biosynthesis, creatine, and cAMP metabolism were also observed [37]. Increased levels of another enzyme involved in fatty acid metabolism (fatty acid binding protein 5, FABP5) were also found in urinary EVs from PCa patients collected after prostatic massage [43]. In this study, the AUC for the prediction of PCa with GS ≥ 6 based on FABP5 was 0.757 (confidence interval 0.570–0.994, *p*-value  =  0.027), whereas the AUC value for the prediction based on serum PSA was 0.593 (confidence interval 0.372–0.815, *p*-value  =  0.42). FABP5 is an enzyme involved in the uptake and transport of fatty acids, that has been previously found to be overexpressed in PCa tissues [81]. Increased levels of this enzyme have been described in serum and tissue samples from PCa patients with lymph node metastasis [82].

Overall, these studies show that the urine metabolic phenotype of PCa patients is significantly different from that of healthy individuals and BPH patients. Taken together, alterations in the levels of metabolites involved in TCA cycle, tryptophan, amino acid, fatty acid, nucleotide, and carbon metabolism have been reported. In general, a significant limitation of these studies has been the sample size, except for the study carried out by Liang et al. where a total of 469 urine samples were analyzed [38]. Therefore, further analyses and validation studies will be necessary to assess the clinical utility of these findings.

### 4.2. Serum Biomarkers

Metabolic dysregulations in TCA cycle, fatty acid, amino acid, purine, histidine, creatine, glycine, and serine, and threonine metabolism have been described when analyzing serum metabolic profile of PCa patients. Particularly, a study conducted by Giskeødegård et al., comparing the serum metabolic profile of 21 BPH and 29 PCa patients, revealed significant changes in fatty acid, choline and amino acid metabolism [49]. In this study, different metabolomics analytical platforms were used to perform the analysis. The combination of the most relevant metabolites identified using the different platforms provided the best classification results, enabling the discrimination of PCa patients and BPH controls with a sensitivity and specificity of 81.5% and 75.2%, respectively. In a different study, Kumar et al. reported a metabolic signature of three metabolites (pyruvate, glycine, and sarcosine) that classified 90.2% of PCa samples (*n* = 70) with 84.8% sensitivity and 92.9% specificity compared with healthy controls (*n* = 32) [47]. Furthermore, Kumar et al., using filtered serum samples (*n* = 210), obtained a model based on five metabolites (alanine, sarcosine, creatinine, glycine, and citrate) that enabled the discrimination of BPH and PCa patients with high accuracy (88.3%) [46]. Finally, Zhao et al., analyzing the metabolic profile of plasma samples from 32 control cases and 32 PCa patients, reported alterations in different metabolic pathways, including amino acid, propanoate, butanoate, and nucleotide metabolism [50]. After evaluation of the predictive value of individual changes, a predictive model combining sarcosine, acetylglycine, and coreximine was reported. However, although a discrete increase in the diagnostic performance (AUC = 0.941; confidence interval 0.812–1) was found when compared with PSA levels (AUC = 0.926; confidence interval 0.851–0.978), this model partially relied on changes in the levels of coreximine, a compound belonging to a family of alkaloids and derivatives, probably from exogenous origin.

Regarding PCa biomarkers associated with disease progression and outcome, different studies, focused on the analysis of PCa serum samples, have been performed trying to identify metabolic alterations that could be useful from this clinical perspective [47,51]. These studies revealed alterations in TCA cycle, lipids, and amino acids metabolism. Lin et al. investigated the correlation between the plasma lipidome and the outcome of 96 castration-resistant PCa (CRPC) patients [51]. A three-lipid signature, comprising ceramide d18:1/24:1, sphingomyelin d18:2/16:0 and phosphatidylcholine 16:0/16:0, was found to be associated with poor prognosis in this study and further validated in an independent cohort of 63 CRPC patients. The results also revealed an association between the lipid signature in the serum of the patients and the overall survival time. Eleven out of the 63 patients of the validation cohort exhibited the three-lipid signature, and their median overall survival time was significantly shorter than those not displaying that signature (11.3 vs. 21.4 months). In another study performed in serum samples, Kumar et al. described a model consisting of three metabolites (alanine, pyruvate and glycine) that allowed the discrimination of low- (*n* = 40) from high-grade (*n* = 30) PCa serum samples with 92.5% sensitivity and 93.3% specificity [47]. Alanine and glycine can be metabolized to a common end product, pyruvate. Increased levels of these two metabolites have also been observed in urine [83] and tissue [84] from PCa patients. Tissue levels of both metabolites have also shown a statistically significant correlation with the GS [85]. Finally, in a study performed by Mondul et al., 200 matched-controls and 200 PCa patients (100 aggressive) were analyzed [52]. The authors reported inverse associations between the risk of aggressive PCa and the levels of glycerophospholipids and fatty acids, inositol-1-phosphate showing the strongest inverse association. On the contrary, aggressive PCa risk was correlated with the levels of α-ketoglutarate, thyroxine, TMAO, and erucoyl-sphingomyelin, while metabolites involved in the metabolism of nucleotides, steroid hormones and tobacco were associated with non-aggressive PCa [52]. In this particular study, although levels of two known nicotine-derived metabolites (cotinine and hydroxycotinine) were found to be associated with non-aggressive PCa, the authors argued that it was unlikely that these changes were related to tobacco smoking as all individuals included in the study were smokers at the time of sample collection. Furthermore, results remained unchanged when adjusting for cigarettes smoked per day, suggesting that cigarette smoking did not strongly influence the results.

Additionally, some of the most recent PCa metabolomics studies based on the analysis of serum samples have aimed to identify metabolic alterations that could provide insights into the risk of developing PCa. These studies were carried out with a significant number of samples in each experimental cohort compared with those focused on the identification of biomarkers for PCa diagnosis and/or prognosis. Thus, Kühn et al. evaluated the association between the levels of pre-diagnostic metabolites and the risk of developing different cancers, including PCa [53]. Serum samples of 310 PCa patients with a median follow-up of 6.83 years were included in the study. High levels of lysophosphatidylcholines were found to be positively correlated to lower PCa risk, while high levels of phosphatidylcholines were associated with increased risk of developing the disease [53]. Schmidt et al. analyzed 1077 healthy and PCa serum samples to assess the risk of developing PCa [54]. In this study, higher citrulline levels were associated with a 27% decreased risk of PCa in the first five years of follow-up but not after longer periods of time [54]. The authors also reported inverse associations between 12 glycerophospholipids and advanced stage disease. In another study, Huang et al. analyzed serum samples from controls (*n* = 200) and PCa patients classified according to their tumor stage (T2: *n* = 71, T3: *n* = 51, T4: *n* = 15), and identified metabolites associated with the risk of being diagnosed with each stage [55]. Histidine and uridine-related metabolites were associated with risk of T2 stage. Glycerophospholipids and primary bile acid lipids showed inverse correlations with T3 stage, while sphingomyelins were positively associated with risk of T3. Secondary bile acid, sex steroids, histamine, and BCAA were associated with T4 risk, while citrate and fumarate were inversely correlated. Finally, a recent study carried out by Andras et al. used serum samples to identify variations in the metabolite levels that could be useful for predicting PCa before biopsy [56]. These authors analyzed 90 samples from patients with suspicion of PCa and derived a predictive score based on six metabolites, that was validated using a subgroup of patients. A cut-off value of 0.528 for the derived score showed good accuracy for PCa prediction before biopsy (AUC = 0.779; confidence interval 0.625–0.876), although not statistically significantly higher than the predictive ability of PSA levels (AUC = 0.793; confidence interval 0.665–0.889). In PCa patients with PSA levels < 10 ng/mL, this score had 80.95% sensitivity and 64.52% specificity for PCa detection at biopsy.

### 4.3. Seminal Fluid Biomarkers

Seminal fluid has a number of advantages over blood and urine in terms of its potential as a source of PCa specific biomarkers. Prostatic constituents are highly enriched in seminal fluid compared with other biofluids. In the last few years, several metabolomics studies have been performed aiming to analyze the metabolic profile of seminal fluid samples from either healthy individuals [57,58,59] or BPH patients [60] and PCa patients to discover metabolic alterations that could be useful for discriminating between both groups. In general, these studies were performed using NMR spectroscopy (*n* = 4) and the sample size of the different cohorts was relatively small. Most of the metabolic alterations identified included changes in the TCA cycle, amino acid, and lipid metabolism. In a preliminary study, Averna et al. found decreased concentrations of citrate in PCa (*n* = 3) compared to BPH (*n* = 1) samples [60]. Similarly, Kline et al. also observed lower citrate levels in PCa samples both when analyzing seminal fluid samples and expressed prostatic secretions (EPS) from 33 healthy volunteers and 28 PCa patients [57]. In this study, authors reported good values for predicting PCa in patients (AUC = 0.81 in seminal fluid, confidence interval 0.60–0.92 and AUC = 0.73 in EPS, confidence interval 0.38–0.90), outperforming the predictive ability of PSA (AUC = 0.61, confidence interval 0.44–0.74) in these samples. Furthermore, using an ELISA assay, Etheridge et al. identified alpha methylacyl A coenzyme racemase (AMACR) as a promising biomarker for PCa diagnosis [58]. Higher levels of this enzyme were detected in seminal fluid samples of PCa patients (*n* = 28) compared with age-matched controls (*n* = 15). AMACR, a key regulator of lipid metabolism, is involved in the peroxisomal and mitochondrial β-oxidation of branched-chain fatty acids. This enzyme had been previously described as an immunohistological marker for PCa diagnosis [86,87], associated with poor prognosis in patients with localized PCa [88] and found to be overexpressed in PCa tissues [89]. Interestingly, AMACR has also been identified as a promising prognostic indicator in other cancer types, including gastric cancer [90] and hepatocellular [91] and nasopharyngeal [92] carcinomas.

Besides seminal fluid, EPS is another biofluid enriched in prostatic material that has shown potential utility for the identification of new PCa disease-specific biomarkers. EPS is obtained in the first void following vigorous DRE or prostatic massage. Given the nature of this biofluid, metabolites present in EPS are usually found at lower concentrations than in seminal fluid, thus requiring the use of highly sensitive detection methods. In 2008, Serkova et al. analyzed EPS samples from 26 healthy volunteers and 52 PCa patients aiming to identify potential metabolites that could contribute to PCa risk assessment [59]. This study revealed that concentrations of citrate, myo-inositol, and spermine were inversely correlated with PCa risk (AUC values of 0.89, 0.87 and 0.79, respectively). However, in a more recent study attempting to validate the role of these metabolites as biomarkers for assessing PCa risk, Roberts et al. found that citrate, spermine, and myo-inositol had minimal predictive ability when analyzing seminal fluid samples [61]. Therefore, further studies using larger cohorts will be required to confirm the utility of seminal fluid and EPS derived biomarkers for PCa diagnosis and prognosis.

## 5. Conclusions and Future Perspectives

The identification and characterization of the metabolic changes accompanying the transformation of benign into malignant prostate cells has led to an increased interest, over the last few years, in the application of metabolomics for identifying clinically relevant biomarkers in this field. Omics approaches, including genomics, proteomics, transcriptomics, and metabolomics, are highly innovative areas of research. One of the major advantages of the omics approaches is their ability to provide information using unbiased large-scale approaches. Among them, metabolomics provides an unprecedented opportunity for understanding the pathophysiological condition of an individual. Metabolites represent the end products of biochemical pathways, and the concentrations of these compounds are extremely sensitive to different alterations. Thus, these metabolic fingerprints can provide useful clues for the characterization of biomarkers associated with the onset and progression of diseases. Furthermore, as metabolomics studies can be performed using biological fluids that could be easily accessible (e.g., serum, plasma, urine, and seminal fluid), it offers a high potential for clinical translatability when compared with other omics approaches.

**Figure 1:**
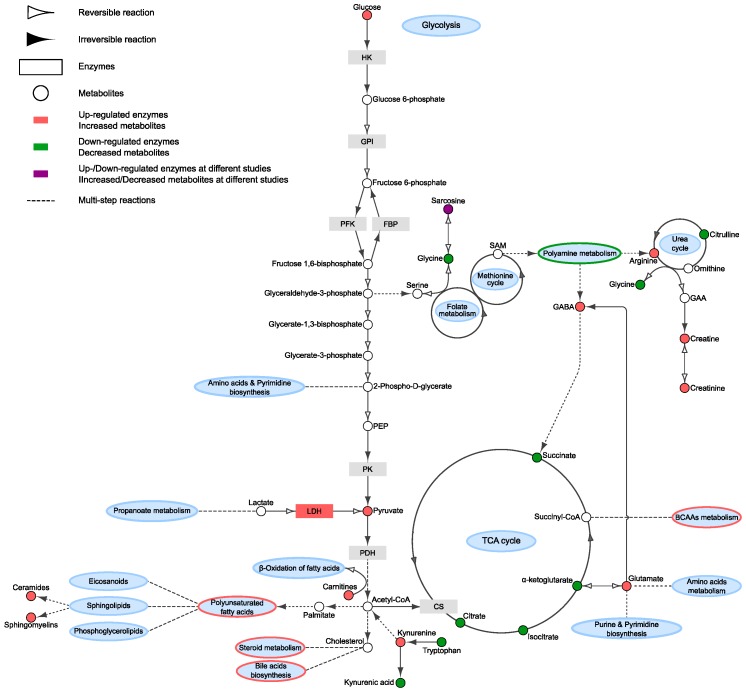
Overview of main metabolic changes described in metabolic-related studies of human biofluids applied to PCa biomarker discovery. BCAA: Branched-chain amino acids; CS: Citrate synthase; FBP1: Fructose-bisphosphatase; GAA: Guanidinoacetate; GABA: Gamma-aminobutyric acid; GPI: Glucose-6-phosphate isomerase; HK2: Hexokinase 2; LDH: Lactate dehydrogenase; PDH: Pyruvate dehydrogenase; PEP: Phosphoenolpyruvate; PFK: Phosphofructokinase; PK: Pyruvate kinase; SAM: S-Adenosyl methionine.

In this manuscript, we aimed to review the main findings described in recent PCa metabolomics studies focused on the analysis of different biofluids (Table 1). Furthermore, a summary of the most significant findings reported in these studies and the connections and interactions between the different metabolic changes described has also been included, aiming to better describe the specific metabolic signature associated to PCa (Figure 1).

Most of the studies included in this review were based on the analysis of blood or urine samples, probably due to their easy accessibility and non-invasiveness. NMR and MS are the two most commonly used analytical platforms in these studies, though other analytical techniques have also been applied to the identification of PCa-related metabolic changes [58,93,94,95]. Although a significant number of studies focused on the identification of biomarkers for PCa diagnosis, some of them also explored the potential of metabolic biomarkers for patient prognosis and PCa risk evaluation.

Overall, these studies have revealed that alterations in TCA cycle, polyamines, glycolysis, one-carbon metabolism, nucleotide synthesis, amino acid, fatty acid, and lipid metabolism are associated with PCa onset and progression. Figure 1 illustrates the main alterations, in terms of metabolic pathways and metabolites, associated with PCa based on current literature.

The results of the different studies provide compelling evidence of the potential of metabolomics strategies for identifying new PCa biomarkers in biofluids that could be of interest from a clinical perspective. The potential of this approach for routine clinical diagnostics is significant since only minimal biological preparation is necessary. Despite the advances achieved in the field of PCa biomarker discovery, intense efforts are still required before metabolite profiling can be implemented in the clinic. So far, the variability in the metabolic alterations reported precludes consistent, universal signatures to be established, showing that a long path is still to be thread toward the full validation and clinical approval of putative new metabolic biomarkers. In this context, it is worth noting that although most of the reviewed studies included the internal validation of the statistical models developed during the study, either for PCa diagnosis or prognosis, a limited number of them included the assessment of the clinical utility of these findings using an external validation cohort of patients. Thus, future studies should include larger sample cohorts from adequately defined and matched groups of samples. In addition, statistical validation of multivariate models would benefit from full external validation. Finally, increased knowledge on the biological significance of potential PCa biomarkers should be assessed through the integration of metabolomics with other biochemical/biological approaches.

## Figures and Tables

**Table 1:** Metabolomics studies focused on the analysis of biofluids to identify clinically relevant prostate cancer (PCa) biomarkers.

Article	Sample	Experimental Approach	Research Aim	Sample Cohort	Main Findings	Validation Cohort
Clos-Garcia et al., 2018 [37]	Urine EVs	UHPLC-MS	Diagnosis	31 × PCa; 14 × BPH	Statistically significant changes in 76 metabolites and 7 enzymes related to urea cycle, TCA cycle, and metabolism of steroid hormone biosynthesis, leukotriene, and prostaglandin, linoleate and purine, glycerophospholipid and tryptophan	No
Liang et al., 2017 [38]	Urine	FPLC-MS/MS	Diagnosis	236 × PCa; 233 × HV	↑ glycocholic acid; hippurate; chenodeoxycholic acid: PCa > HV ↓ taurocholic acid; 5-hydroxy-l-tryptophan: PCa < HV	No
Gkotsos et al., 2017 [39]	Urine	UPLC-MS/MS	Diagnosis	52 × PCa, 49 × HV	↓ kynurenic acid: PCa < HV	No
Struck-Lewicka et al., 2015 [40]	Urine	HPLC-TOF-MS; GC-QqQ-MS	Diagnosis	32 × PCa; 32 × HV	Statistically significant changes in 82 metabolites related to amino acid, organic acids, sphingolipids, fatty acids, and carbohydrates metabolism	No
Fernández-Peralbo et al., 2016 [41]	Urine	LC-QTOF-MS/MS	Diagnosis	43 × PCa; 29 × HV	↑ 7-methylguanine: PCa > HV ↓ Statistically significant changes in 27 metabolites related to amino acid metabolism: PCa < HV	19 × PCa; 13 × HV
Puhka et al., 2017 [42]	Urine EVs; Plasma EVs	UPLC-MS/MS	Diagnosis	3 × PCa pre-prostatectomy; 3 × PCa post-prostatectomy; 3 × HV	↓ glucoronate; isobutyryl-L-carnitine; D-Ribose-5-phosphate: pre- < post-prostatectomy and HV	No
Fujita et al., 2017 [43]	Urine EVs	iTRAQ; LC-MS/MS	Diagnosis and prognosis	12 × PCa (6 × HG PCa; 6 × LG PCa); 6 × HV	↑ FABP5: PCa > HV ↑ FABP5; GRN; AMBP; CHMP4A; CHMP4C associated with higher GS	18 × PCa (6 × HG; 12 × LG); 11 × HV
Perez-Rambla et al., 2017 [44]	Urine	^1^H-NMR	Diagnosis	64 × PCa; 51 × BPH	↑ BCAAs; glutamate; pseudouridine: PCa > BPH ↓ glycine; dimethylglycine; fumarate; 4-imidazole-acetate: PCa < BPH	No
Davalieva et al., 2015 [45]	Urine	2D-DIGE-MS	Diagnosis	8 × PCa; 16 × BPH	↑ AMBP: PCa > BPH ↓ HP: PCa < BPH	16 × PCa; 16 × BPH
Heger et al., 2015 [48]	Urine	2D-DIGE; MALDI-TOF-MS	Diagnosis	15 × HG PCa; 15 × LG PCa	↑ CDK6; M2BP; LDHC: HG PCa > LG PCa	No
Kumar et al., 2016 [46]	Serum	^1^H-NMR	Diagnosis	75 × PCa; 70 × BPH; 65 x HV	↑ alanine; sarcosine; creatine; creatinine: PCa > BPH and HV ↑ pyruvate; 3-methylhistidine; xanthine; hypoxanthine: BPH and PCa > HV ↓ glycine: PCa < HV ↓ citrate: PCa < BPH and HV	No
Kumar et al., 2015 [47]	Serum	^1^H-NMR	Diagnosis and prognosis	21 × HG PCa; 28 × LG PCa; 22 × HV	↑ alanine; sarcosine: LG PCa > HG PCa and HV ↑ pyruvate: LG PCa and HG PCa > HV ↓ glycine: LG PCa and HG PCa < HV	9 × HG PCa; 12 × LG PCa; 12 × HV
Giskeødegård et al., 2015 [49]	Plasma/Serum	^1^H-NMR; UPLC-MS/MS; GC-MS	Diagnosis	29 × PCa; 21 × BPH	↑ decanoylcarnitine (c10); tetradecenoylcarnitine (c14:1); octanoyl-carnitine (c8); dimethylsulfone; phenylalanine; lysine: PCa > BPH ↓ phosphatidylcholine diacyl (c34:4); lipid -(CH2)n-CH2-CH2-CO: PCa < BPH	No
Zhao et al., 2017 [50]	Plasma	UPLC-MS/MS	Diagnosis	32 × PCa; 32 × HV	Statistically significant changes in 19 metabolites related to amino acid, nucleotide, butanoate and propionate metabolism	No
Lin et al., 2017 [51]	Plasma	LC-MS/MS	Prognosis	96 × CRPC	↑ ceramide d18:1/24:1; sphingomyelin d18:2/16:0; phosphatidylcholine 16:0/16:0 correlated with shorter overall survival	63 × CRPC
Mondul et al., 2015 [52]	Serum	UHPLC-MS; GC-MS	PCa risk Prognosis	100 × HG PCa; 100 × LG PCa; 200 × HV	Statistically significant changes in 22 metabolites related to lipid and amino acid metabolism associated with overall PCa risk Statistically significant changes in 14 metabolites related to TCA cycle and lipid metabolism associated with HG PCa Statistically significant changes in 34 metabolites related to lipid, amino acid and nucleotide metabolism associated with LG PCa	No
Kühn et al., 2016 [53]	Plasma	LC-MS/MS; FIA-MS/MS	PCa risk	310 × PCa; 774 × HV	↑ Phosphatidylcholine (PC) associated with higher risk of PCa ↑ lysoPC associated with lower risk of PCa	No
Schmidt et al., 2017 [54]	Plasma	QqQ-MS	PCa risk Prognosis	1077 × PCa; 1077 × HV 208 × advanced PCa; 456 × localized PCa	Statistically significant changes in 14 metabolites related to lipid and amino acid metabolism associated with overall PCa risk 12 glycerophospholipids inversely associated with risk of advanced PCa	No No
Huang et al., 2017 [55]	Serum	UHPLC-MS; GC-MS	PCa risk	71 × PCa T2 stage; 51 × PCa T3 stage; 15 × PCa T4 stage; 200 × HV	Statistically significant changes in 8 metabolites related to histidine and uridine metabolism associated with PCa T2 risk. Statistically significant changes in 12 metabolites related to fatty acid and primary bile acid metabolism associated with PCa T3 risk Statistically significant changes in 16 metabolites related to TCA, BCAA secondary bile acid, sex steroids and histamine metabolism associated with PCa T4 risk.	No
Andras et al., 2017 [56]	Serum	HPLC-ESI-QTOF-MS	Prediction	59 × patients with high PSA levels	6 metabolites involved in lipid, purine and tryptophan metabolism predictive for prostate biopsy outcome	31 × patients with high PSA levels
Kline et al., 2006 [57]	Seminal fluid; Prostatic secretion	^1^H-NMR	Diagnosis	28 × PCa; 33 × HV	↓ citrate: PCa < HV	No
Etheridge et al., 2018 [58]	Seminal fluid	ELISA	Diagnosis	28 × PCa; 15 × HV	↑ AMACR: PCa > HV	No
Serkova et al., 2008 [59]	Prostatic secretion	^1^H-NMR	Prediction PCa risk	52 × PCa; 26 × HV	↓ citrate; myo-inositol; spermine shown highly predictive of PCa and inversely associated with PCa risk	No
Averna et al., 2005 [60]	Seminal fluid	^1^H-NMR	Diagnosis	3 × PCa; 1 × BPH; 4 × HV	↓ citrate: PCa < BPH	No
Roberts et al., 2017 [61]	Seminal fluid	^1^H-NMR	Prediction	98 × PCa; 53 × HV	Statistically significant changes in choline, valine and leucine,	No

^1^H-NMR: Proton nuclear magnetic resonance spectroscopy; 2D-DIGE-MS: Two dimensional–difference gel electrophoresis–mass spectrometry; BCAA: Branched-chain amino acids; BPH: Benign Prostatic Hyperplasia; CRPC: Metastatic castration-resistant prostate cancer; ELISA: Enzyme-linked immunosorbent assay; EV: Extracellular vesicles; FIA-MS/MS: Flow injection analysis–tandem mass spectrometry; FPLC-MS: Fast ultra-high performance liquid chromatography–mass spectrometry; GC-MS: Gas chromatography–mass spectrometry; GC-QqQ-MS: Gas chromatography–triple quadrupole–mass spectrometry; HG: High-grade (GS ≥ 8); HPLC-ESI-QTOF-MS: High performance liquid chromatography–electrospray ionization–quadrupole time of flight–mass spectrometry; HPLC-TOF-MS: High performance liquid chromatography–time of flight–mass spectrometry; HV: Healthy Volunteers; iTRAQ: Isobaric tag for relative and absolute quantification; LC-MS: Liquid chromatography–mass spectrometry; LC-MS/MS: Liquid chromatography–tandem mass spectrometry; LG: Low-grade (GS ≤ 7); MALDI-TOF-MS: Matrix-assisted laser desorption ionization–time of flight–mass spectrometry; PCa: Prostate Cancer; PM: prostatic massage; QqQ-MS: Triple quadrupole–mass spectrometry; T: Stage; TCA: Tricarboxylic acid; UHPLC-MS: Ultra high performance liquid chromatography–mass spectrometry; UPLC-MS/MS: Ultra performance liquid chromatography–tandem mass spectrometry.

## Abbreviations

The following abbreviations are used in this manuscript:

^1^H-NMR	Proton nuclear magnetic resonance spectroscopy
2D-DIGE-MS	Two dimensional–difference gel electrophoresis–mass spectrometry
AUC	Area under the curve
BCAA	Branched-chain amino acids
BPH	Benign prostatic hyperplasia
CRPC	Castration-resistant prostate cancer
CS	Citrate synthase
DRE	Digital rectal examination
ELISA	Enzyme-linked immunosorbent assay
EPS	Expressed prostatic secretions
EV	Extracellular vesicles
FBP	Fructose-bisphosphatase
FIA-MS/MS	Flow injection analysis–tandem mass spectrometry
FPLC-MS	Fast ultra-high-performance liquid chromatography–mass spectrometry
GAA	Guanidinoacetate
GABA	Gamma-aminobutyric acid
GPI	Glucose-6-phosphate isomerase
GS	Gleason Score
GC-MS	Gas chromatography–mass spectrometry
GC-QqQ-MS	Gas chromatography–triple quadrupole–mass spectrometry
HG	High-grade (GS ≥ 8)
HK2	Hexokinase 2
HPLC-ESI-QTOF-MS	High performance liquid chromatography–electrospray ionization–quadrupole time of flight–mass spectrometry
HPLC-TOF-MS	High performance liquid chromatography–time of flight–mass spectrometry
HV	Healthy Volunteers
iTRAQ	Isobaric tag for relative and absolute quantification
LC-MS	Liquid chromatography–mass spectrometry
LC-MS/MS	Liquid chromatography–tandem mass spectrometry
LDH	Lactate dehydrogenase
LG	Low-grade (GS ≤ 7)
MALDI-TOF-MS	Matrix-assisted laser desorption ionization–time of flight–mass spectrometry
MS	Mass spectroscopy
NMR	Nuclear magnetic resonance
QqQ-MS:	Triple quadrupole–mass spectrometry
PCa	Prostate cancer
PDH	Pyruvate dehydrogenase
PEP	Phosphoenolpyruvate
PFK	Phosphofructokinase
PK	Pyruvate kinase
PM	Prostatic massage
PSA	Prostate specific antigen
SAM	S-Adenosyl methionine
T	Stage
TCA	Tricarboxylic acid
TMAO	Trimethylamine N-oxide
TRUS	Trans-rectal ultrasound
UHPLC-MS	Ultra-high-performance liquid chromatography–mass spectrometry
UPLC-MS/MS	Ultra performance liquid chromatography–tandem mass spectrometry

## References

[1] Bray F., Ferlay J., Soerjomataram I., Siegel R.L., Torre L.A., Jemal A. (2018). Global cancer statistics 2018: GLOBOCAN estimates of incidence and mortality worldwide for 36 cancers in 185 countries. CA Cancer J. Clin..

[2] Schoenfield L., Jones J.S., Zippe C.D., Reuther A.M., Klein E., Zhou M., Magi-Galluzzi C. (2007). The incidence of high-grade prostatic intraepithelial neoplasia and atypical glands suspicious for carcinoma on first-time saturation needle biopsy, and the subsequent risk of cancer. BJU Int..

[3] Offermann A., Hohensteiner S., Kuempers C., Ribbat-Idel J., Schneider F., Becker F., Hupe M.C., Duensing S., Merseburger A.S., Kirfel J. (2017). Prognostic Value of the New Prostate Cancer International Society of Urological Pathology Grade Groups. Front. Med..

[4] Chistiakov D.A., Myasoedova V.A., Grechko A.V., Melnichenko A.A., Orekhov A.N. (2018). New biomarkers for diagnosis and prognosis of localized prostate cancer. Semin. Cancer Biol..

[5] Gordetsky J., Epstein J. (2016). Grading of prostatic adenocarcinoma: Current state and prognostic implications. Diagn. Pathol..

[6] Foley R.W., Maweni R.M., Gorman L., Murphy K., Lundon D.J., Durkan G., Power R., O’Brien F., O’Malley K.J., Galvin D.J. (2016). European Randomised Study of Screening for Prostate Cancer (ERSPC) risk calculators significantly outperform the Prostate Cancer Prevention Trial (PCPT) 2.0 in the prediction of prostate cancer: A multi-institutional study. BJU Int..

[7] Nam R.K., Satkunasivam R., Chin J.L., Izawa J., Trachtenberg J., Rendon R., Bell D., Singal R., Sherman C., Sugar L. (2017). Next-generation prostate cancer risk calculator for primary care physicians. Can. Urol. Assoc. J..

[8] Loeb S., Partin A.W. (2011). Review of the Literature: PCA3 for Prostate Cancer Risk Assessment and Prognostication. Rev. Urol..

[9] Sanhueza C., Kohli M. (2018). Clinical and Novel Biomarkers in the Management of Prostate Cancer. Curr. Treat. Options Oncol..

[10] (2014). Biomarkers PCA3 and TMPRSS2-ERG: Better together: Prostate cancer. Nat. Rev. Urol..

[11] Perner S., Mosquera J.-M., Demichelis F., Hofer M.D., Paris P.L., Simko J., Collins C., Bismar T.A., Chinnaiyan A.M., De Marzo A.M. (2007). TMPRSS2-ERG Fusion Prostate Cancer: An Early Molecular Event Associated with Invasion. Am. J. Surg. Pathol..

[12] Barbieri C.E., Demichelis F., Rubin M.A. (2012). Molecular genetics of prostate cancer: Emerging appreciation of genetic complexity. Histopathology.

[13] Tomlins S.A. (2005). Recurrent Fusion of TMPRSS2 and ETS Transcription Factor Genes in Prostate. Cancer Sci..

[14] Ferro M., Buonerba C., Terracciano D., Lucarelli G., Cosimato V., Bottero D., Deliu V.M., Ditonno P., Perdonà S., Autorino R. (2016). Biomarkers in localized prostate cancer. Future Oncol..

[15] Hendriks R.J., van Oort I.M., Schalken J.A. (2017). Blood-based and urinary prostate cancer biomarkers: A review and comparison of novel biomarkers for detection and treatment decisions. Prostate Cancer Prostatic Dis..

[16] Khan A., Choi S.A., Na J., Pamungkas A.D., Jung K.J., Jee S.H., Park Y.H. (2019). Non-invasive Serum Metabolomic Profiling Reveals Elevated Kynurenine Pathway’s Metabolites in Humans with Prostate Cancer. J. Proteome Res..

[17] Andersen M.K., Rise K., Giskeødegård G.F., Richardsen E., Bertilsson H., Størkersen Ø., Bathen T.F., Rye M., Tessem M.-B. (2018). Integrative metabolic and transcriptomic profiling of prostate cancer tissue containing reactive stroma. Sci. Rep..

[18] Fujita K., Nonomura N. (2018). Urinary biomarkers of prostate cancer. Int. J. Urol..

[19] Kumar D., Gupta A., Nath K. (2016). NMR-based metabolomics of prostate cancer: A protagonist in clinical diagnostics. Expert Rev. Mol. Diagn..

[20] Holmes E., Wilson I.D., Nicholson J.K. (2008). Metabolic phenotyping in health and disease. Cell.

[21] Warburg O. (1956). On the origin of cancer cells. Science.

[22] Vander Heiden M.G., DeBerardinis R.J. (2017). Understanding the Intersections between Metabolism and Cancer Biology. Cell.

[23] Hanahan D., Weinberg R.A. (2011). Hallmarks of cancer: The next generation. Cell.

[24] Pavlova N.N., Thompson C.B. (2016). The Emerging Hallmarks of Cancer Metabolism. Cell Metab..

[25] Levine A.J., Puzio-Kuter A.M. (2010). The control of the metabolic switch in cancers by oncogenes and tumor suppressor genes. Science.

[26] Ward P.S., Patel J., Wise D.R., Abdel-Wahab O., Bennett B.D., Coller H.A., Cross J.R., Fantin V.R., Hedvat C.V., Perl A.E. (2010). The common feature of leukemia-associated IDH1 and IDH2 mutations is a neomorphic enzyme activity converting alpha-ketoglutarate to 2-hydroxyglutarate. Cancer Cell.

[27] Hipp S.J., Steffen-Smith E.A., Patronas N., Herscovitch P., Solomon J.M., Bent R.S., Steinberg S.M., Warren K.E. (2012). Molecular imaging of pediatric brain tumors: Comparison of tumor metabolism using 18F-FDG-PET and MRSI. J. Neurooncol..

[28] Zhan H., Ciano K., Dong K., Zucker S. (2015). Targeting glutamine metabolism in myeloproliferative neoplasms. Blood Cells Mol. Dis..

[29] Sutinen E., Nurmi M., Roivainen A., Varpula M., Tolvanen T., Lehikoinen P., Minn H. (2004). Kinetics of [(11)C]choline uptake in prostate cancer: A PET study. Eur. J. Nuclear Med. Mol. Imaging.

[30] Srivastava A., Creek D.J. (2018). Discovery and Validation of Clinical Biomarkers of Cancer: A Review Combining Metabolomics and Proteomics. Proteomics.

[31] Zhang A., Sun H., Yan G., Wang P., Wang X. (2015). Metabolomics for Biomarker Discovery: Moving to the Clinic. Biomed. Res. Int..

[32] Mirnaghi F.S., Caudy A.A. (2014). Challenges of analyzing different classes of metabolites by a single analytical method. Bioanalysis.

[33] Wolfender J.-L., Marti G., Thomas A., Bertrand S. (2015). Current approaches and challenges for the metabolite profiling of complex natural extracts. J. Chromatogr. A.

[34] Alonso A., Marsal S., Julià A. (2015). Analytical methods in untargeted metabolomics: State of the art in 2015. Front. Bioeng. Biotechnol..

[35] Bingol K., Brüschweiler R. (2015). Two elephants in the room: New hybrid nuclear magnetic resonance and mass spectrometry approaches for metabolomics. Curr. Opin. Clin. Nutr. Metab. Care.

[36] Fuhrer T., Zamboni N. (2015). High-throughput discovery metabolomics. Curr. Opin. Biotechnol..

[37] Clos-Garcia M., Loizaga-Iriarte A., Zuñiga-Garcia P., Sánchez-Mosquera P., Rosa Cortazar A., González E., Torrano V., Alonso C., Pérez-Cormenzana M., Ugalde-Olano A. (2018). Metabolic alterations in urine extracellular vesicles are associated to prostate cancer pathogenesis and progression. J. Extracell. Vesicles.

[38] Liang Q., Liu H., Xie L., Li X., Zhang A.-H. (2017). High-throughput metabolomics enables biomarker discovery in prostate cancer. RSC Adv..

[39] Gkotsos G., Virgiliou C., Lagoudaki I., Sardeli C., Raikos N., Theodoridis G., Dimitriadis G. (2017). The Role of Sarcosine, Uracil, and Kynurenic Acid Metabolism in Urine for Diagnosis and Progression Monitoring of Prostate Cancer. Metabolites.

[40] Struck-Lewicka W., Kordalewska M., Bujak R., Yumba Mpanga A., Markuszewski M., Jacyna J., Matuszewski M., Kaliszan R., Markuszewski M.J. (2015). Urine metabolic fingerprinting using LC–MS and GC–MS reveals metabolite changes in prostate cancer: A pilot study. J. Pharm. Biomed. Anal..

[41] Fernández-Peralbo M.A., Gómez-Gómez E., Calderón-Santiago M., Carrasco-Valiente J., Ruiz-García J., Requena-Tapia M.J., Luque de Castro M.D., Priego-Capote F. (2016). Prostate Cancer Patients–Negative Biopsy Controls Discrimination by Untargeted Metabolomics Analysis of Urine by LC-QTOF: Upstream Information on Other Omics. Sci. Rep..

[42] Puhka M., Takatalo M., Nordberg M.-E., Valkonen S., Nandania J., Aatonen M., Yliperttula M., Laitinen S., Velagapudi V., Mirtti T. (2017). Metabolomic Profiling of Extracellular Vesicles and Alternative Normalization Methods Reveal Enriched Metabolites and Strategies to Study Prostate Cancer-Related Changes. Theranostics.

[43] Fujita K., Kume H., Matsuzaki K., Kawashima A., Ujike T., Nagahara A., Uemura M., Miyagawa Y., Tomonaga T., Nonomura N. (2017). Proteomic analysis of urinary extracellular vesicles from high Gleason score prostate cancer. Sci. Rep..

[44] Pérez-Rambla C., Puchades-Carrasco L., García-Flores M., Rubio-Briones J., López-Guerrero J.A., Pineda-Lucena A. (2017). Non-invasive urinary metabolomic profiling discriminates prostate cancer from benign prostatic hyperplasia. Metabolomics.

[45] Davalieva K., Kostovska I.M., Kiprijanovska S., Markoska K., Kubelka-Sabit K., Filipovski V., Stavridis S., Stankov O., Komina S., Petrusevska G. (2015). Proteomics analysis of malignant and benign prostate tissue by 2D DIGE/MS reveals new insights into proteins involved in prostate cancer: Proteomics Analysis of Prostate Cancer. Prostate.

[46] Kumar D., Gupta A., Mandhani A., Sankhwar S.N. (2016). NMR spectroscopy of filtered serum of prostate cancer: A new frontier in metabolomics: Metabolomics of Prostate Cancer. Prostate.

[47] Kumar D., Gupta A., Mandhani A., Sankhwar S.N. (2015). Metabolomics-Derived Prostate Cancer Biomarkers: Fact or Fiction?. J. Proteome Res..

[48] Heger Z., Michalek P., Guran R., Cernei N., Duskova K., Vesely S., Anyz J., Stepankova O., Zitka O., Adam V. (2015). Differences in urinary proteins related to surgical margin status after radical prostatectomy. Oncol. Rep..

[49] Giskeødegård G.F., Hansen A.F., Bertilsson H., Gonzalez S.V., Kristiansen K.A., Bruheim P., Mjøs S.A., Angelsen A., Bathen T.F., Tessem M.-B. (2015). Metabolic markers in blood can separate prostate cancer from benign prostatic hyperplasia. Br. J. Cancer.

[50] Zhao Y., Lv H., Qiu S., Gao L., Ai H. (2017). Plasma metabolic profiling and novel metabolite biomarkers for diagnosing prostate cancer. RSC Adv..

[51] Lin H.-M., Mahon K.L., Weir J.M., Mundra P.A., Spielman C., Briscoe K., Gurney H., Mallesara G., Marx G., Stockler M.R. (2017). A distinct plasma lipid signature associated with poor prognosis in castration-resistant prostate cancer: Prognostic lipid signature in metastatic prostate cancer. Int. J. Cancer.

[52] Mondul A.M., Moore S.C., Weinstein S.J., Karoly E.D., Sampson J.N., Albanes D. (2015). Metabolomic analysis of prostate cancer risk in a prospective cohort: The alpha-tocopherol, beta-carotene cancer prevention (ATBC) study: Serum Metabolomics Profiling of Prostate Cancer Risk. Int. J. Cancer.

[53] Kühn T., Floegel A., Sookthai D., Johnson T., Rolle-Kampczyk U., Otto W., von Bergen M., Boeing H., Kaaks R. (2016). Higher plasma levels of lysophosphatidylcholine 18:0 are related to a lower risk of common cancers in a prospective metabolomics study. BMC Med..

[54] Schmidt J.A., Fensom G.K., Rinaldi S., Scalbert A., Appleby P.N., Achaintre D., Gicquiau A., Gunter M.J., Ferrari P., Kaaks R. (2017). Pre-diagnostic metabolite concentrations and prostate cancer risk in 1077 cases and 1077 matched controls in the European Prospective Investigation into Cancer and Nutrition. BMC Med..

[55] Huang J., Mondul A.M., Weinstein S.J., Karoly E.D., Sampson J.N., Albanes D. (2017). Prospective serum metabolomic profile of prostate cancer by size and extent of primary tumor. Oncotarget.

[56] Andras I., Crisan N., Vesa S., Rahota R., Romanciuc F., Lazar A., Socaciu C., Matei D.-V., de Cobelli O., Bocsan I.-S. (2017). Serum metabolomics can predict the outcome of first systematic transrectal prostate biopsy in patients with PSA <10 ng/mL. Future Oncol..

[57] Kline E.E., Treat E.G., Averna T.A., Davis M.S., Smith A.Y., Sillerud L.O. (2006). Citrate Concentrations in Human Seminal Fluid and Expressed Prostatic Fluid Determined via 1H Nuclear Magnetic Resonance Spectroscopy Outperform Prostate Specific Antigen in Prostate Cancer Detection. J. Urol..

[58] Etheridge T., Straus J., Ritter M.A., Jarrard D.F., Huang W. (2018). Semen AMACR protein as a novel method for detecting prostate cancer. Urol. Oncol..

[59] Serkova N.J., Gamito E.J., Jones R.H., O’Donnell C., Brown J.L., Green S., Sullivan H., Hedlund T., Crawford E.D. (2008). The metabolites citrate, myo-inositol, and spermine are potential age-independent markers of prostate cancer in human expressed prostatic secretions. Prostate.

[60] Averna T., Kline E., Smith A., Sillerud L. (2005). A decrease in 1h nuclear magnetic resonance spectroscopically determined citrate in human seminal fluid accompanies the development of prostate adenocarcinoma. J. Urol..

[61] Roberts M.J., Richards R.S., Chow C.W.K., Buck M., Yaxley J., Lavin M.F., Schirra H.J., Gardiner R.A. (2017). Seminal plasma enables selection and monitoring of active surveillance candidates using nuclear magnetic resonance-based metabolomics: A preliminary investigation. Prostate Int..

[62] Eidelman E., Twum-Ampofo J., Ansari J., Siddiqui M.M. (2017). The Metabolic Phenotype of Prostate Cancer. Front. Oncol..

[63] Lima A., Araújo A., Pinto J., Jerónimo C., Henrique R., Bastos M., Carvalho M., Guedes de Pinho P. (2018). GC-MS-Based Endometabolome Analysis Differentiates Prostate Cancer from Normal Prostate Cells. Metabolites.

[64] Giunchi F., Fiorentino M., Loda M. (2018). The Metabolic Landscape of Prostate Cancer. Eur. Urol. Oncol..

[65] Sadeghi R.N., Karami-Tehrani F., Salami S. (2015). Targeting prostate cancer cell metabolism: Impact of hexokinase and CPT-1 enzymes. Tumour Biol..

[66] Twum-Ampofo J., Fu D.-X., Passaniti A., Hussain A., Siddiqui M.M. (2016). Metabolic targets for potential prostate cancer therapeutics. Curr. Opin. Oncol..

[67] Awwad H.M., Geisel J., Obeid R. (2012). The role of choline in prostate cancer. Clin. Biochem..

[68] Sreekumar A., Poisson L.M., Rajendiran T.M., Khan A.P., Cao Q., Yu J., Laxman B., Mehra R., Lonigro R.J., Li Y. (2009). Metabolomic profiles delineate potential role for sarcosine in prostate cancer progression. Nature.

[69] Giskeødegård G.F., Bertilsson H., Selnæs K.M., Wright A.J., Bathen T.F., Viset T., Halgunset J., Angelsen A., Gribbestad I.S., Tessem M.-B. (2013). Spermine and Citrate as Metabolic Biomarkers for Assessing Prostate Cancer Aggressiveness. PLoS ONE.

[70] Zabala-Letona A., Arruabarrena-Aristorena A., Martín-Martín N., Fernandez-Ruiz S., Sutherland J.D., Clasquin M., Tomas-Cortazar J., Jimenez J., Torres I., Quang P. (2017). mTORC1-dependent AMD1 regulation sustains polyamine metabolism in prostate cancer. Nature.

[71] Wu D., Ni J., Beretov J., Cozzi P., Willcox M., Wasinger V., Walsh B., Graham P., Li Y. (2017). Urinary biomarkers in prostate cancer detection and monitoring progression. Crit. Rev. Oncol. Hematol..

[72] Amobi A., Qian F., Lugade A.A., Odunsi K. (2017). Tryptophan Catabolism and Cancer Immunotherapy Targeting IDO Mediated Immune Suppression. Adv. Exp. Med. Biol..

[73] Santhanam S., Alvarado D.M., Ciorba M.A. (2016). Therapeutic targeting of inflammation and tryptophan metabolism in colon and gastrointestinal cancer. Transl. Res..

[74] Khan A.P., Rajendiran T.M., Bushra A., Asangani I.A., Athanikar J.N., Yocum A.K., Mehra R., Siddiqui J., Palapattu G., Wei J.T. (2013). The Role of Sarcosine Metabolism in Prostate Cancer Progression. Neoplasia.

[75] Ankerst D.P., Liss M., Zapata D., Hoefler J., Thompson I.M., Leach R.J. (2015). A case control study of sarcosine as an early prostate cancer detection biomarker. BMC Urol..

[76] Dereziński P., Klupczynska A., Sawicki W., Pałka J.A., Kokot Z.J. (2017). Amino Acid Profiles of Serum and Urine in Search for Prostate Cancer Biomarkers: A Pilot Study. Int. J. Med. Sci..

[77] Locasale J.W. (2013). Serine, glycine and one-carbon units: Cancer metabolism in full circle. Nat. Rev. Cancer.

[78] Koslowski M., Türeci O., Bell C., Krause P., Lehr H.-A., Brunner J., Seitz G., Nestle F.O., Huber C., Sahin U. (2002). Multiple splice variants of lactate dehydrogenase C selectively expressed in human cancer. Cancer Res..

[79] Kong L., Du W., Cui Z., Wang L., Yang Z., Zhang H., Lin D. (2016). Expression of lactate dehydrogenase C in MDA-MB-231 cells and its role in tumor invasion and migration. Mol. Med. Rep..

[80] Merchant M.L., Rood I.M., Deegens J.K.J., Klein J.B. (2017). Isolation and characterization of urinary extracellular vesicles: Implications for biomarker discovery. Nat. Rev. Nephrol..

[81] Myers J.S., von Lersner A.K., Sang Q.-X.A. (2016). Proteomic Upregulation of Fatty Acid Synthase and Fatty Acid Binding Protein 5 and Identification of Cancer- and Race-Specific Pathway Associations in Human Prostate Cancer Tissues. J. Cancer.

[82] Pang J., Liu W.-P., Liu X.-P., Li L.-Y., Fang Y.-Q., Sun Q.-P., Liu S.-J., Li M.-T., Su Z.-L., Gao X. (2010). Profiling protein markers associated with lymph node metastasis in prostate cancer by DIGE-based proteomics analysis. J. Proteome Res..

[83] Wu H., Liu T., Ma C., Xue R., Deng C., Zeng H., Shen X. (2011). GC/MS-based metabolomic approach to validate the role of urinary sarcosine and target biomarkers for human prostate cancer by microwave-assisted derivatization. Anal. Bioanal. Chem..

[84] Kami K., Fujimori T., Sato H., Sato M., Yamamoto H., Ohashi Y., Sugiyama N., Ishihama Y., Onozuka H., Ochiai A. (2013). Metabolomic profiling of lung and prostate tumor tissues by capillary electrophoresis time-of-flight mass spectrometry. Metabolomics.

[85] McDunn J.E., Li Z., Adam K.-P., Neri B.P., Wolfert R.L., Milburn M.V., Lotan Y., Wheeler T.M. (2013). Metabolomic signatures of aggressive prostate cancer. Prostate.

[86] Jiang Z., Woda B.A. (2004). Diagnostic utility of alpha-methylacyl CoA racemase (P504S) on prostate needle biopsy. Adv. Anat. Pathol..

[87] Zhou M., Jiang Z., Epstein J.I. (2003). Expression and diagnostic utility of alpha-methylacyl-CoA-racemase (P504S) in foamy gland and pseudohyperplastic prostate cancer. Am. J. Surg. Pathol..

[88] Box A., Alshalalfa M., Hegazy S.A., Donnelly B., Bismar T.A. (2016). High alpha-methylacyl-CoA racemase (AMACR) is associated with ERG expression and with adverse clinical outcome in patients with localized prostate cancer. Tumour Biol..

[89] Alinezhad S., Väänänen R.-M., Ochoa N.T., Vertosick E.A., Bjartell A., Boström P.J., Taimen P., Pettersson K. (2016). Global expression of AMACR transcripts predicts risk for prostate cancer—A systematic comparison of AMACR protein and mRNA expression in cancerous and noncancerous prostate. BMC Urol..

[90] Mroz A., Kiedrowski M., Lewandowski Z. (2013). α-Methylacyl-CoA racemase (AMACR) in gastric cancer: Correlation with clinicopathologic data and disease-free survival. Appl. Immunohistochem. Mol. Morphol..

[91] Xu B., Cai Z., Zeng Y., Chen L., Du X., Huang A., Liu X., Liu J. (2014). α-Methylacyl-CoA racemase (AMACR) serves as a prognostic biomarker for the early recurrence/metastasis of HCC. J. Clin. Pathol..

[92] Lee Y.-E., He H.-L., Lee S.-W., Chen T.-J., Chang K.-Y., Hsing C.-H., Li C.-F. (2014). AMACR overexpression as a poor prognostic factor in patients with nasopharyngeal carcinoma. Tumour Biol..

[93] Da Costa I.A., Hennenlotter J., Stühler V., Kühs U., Scharpf M., Todenhöfer T., Stenzl A., Bedke J. (2018). Transketolase like 1 (TKTL1) expression alterations in prostate cancer tumorigenesis. Urol. Oncol..

[94] Kojima Y., Yoneyama T., Hatakeyama S., Mikami J., Sato T., Mori K., Hashimoto Y., Koie T., Ohyama C., Fukuda M. (2015). Detection of Core2 β-1,6-*N*-Acetylglucosaminyltransferase in Post-Digital Rectal Examination Urine Is a Reliable Indicator for Extracapsular Extension of Prostate Cancer. PLoS ONE.

[95] Sato T., Yoneyama T., Tobisawa Y., Hatakeyama S., Yamamoto H., Kojima Y., Mikami J., Mori K., Hashimoto Y., Koie T. (2016). Core 2 β-1, 6-N-acetylglucosaminyltransferase-1 expression in prostate biopsy specimen is an indicator of prostate cancer aggressiveness. Biochem. Biophys. Res. Commun..

